# (Formato-κ^2^ *O*,*O*′)bis­(1,10-phenanthroline-κ^2^ *N*,*N*′)manganese(II) perchlorate

**DOI:** 10.1107/S1600536809049277

**Published:** 2009-11-21

**Authors:** Jun Zhao, Xue-Gang Zheng, Zong-Zhi Hu

**Affiliations:** a College of Mechanical & Material Engineering, Functional Materials Research Institute, China Three Gorges University, Yichang 443002, People’s Republic of China; b Lanzhou Institute of Biological Products, Lanzhou 730046, People’s Republic of China

## Abstract

In the title complex, [Mn(CHO_2_)(C_12_H_8_N_2_)_2_]ClO_4_, the Mn^II^ cation is chelated by two 1,10-phenanthroline (phen) ligands and one formate anion in a distorted MnN_4_O_2_ octa­hedral geometry. The two phen planes are oriented at a dihedral angle of 57.48 (11)°. The perchlorate anion links with the Mn complex cation *via* weak C—H⋯O hydrogen bonding.

## Related literature

For general background to manganese(II)–phen complexes and related structures, see: Zhu *et al.* (2008 ▶); Hao *et al.* (2008 ▶); Zhang (2004 ▶); Xu & Xu (2005 ▶).
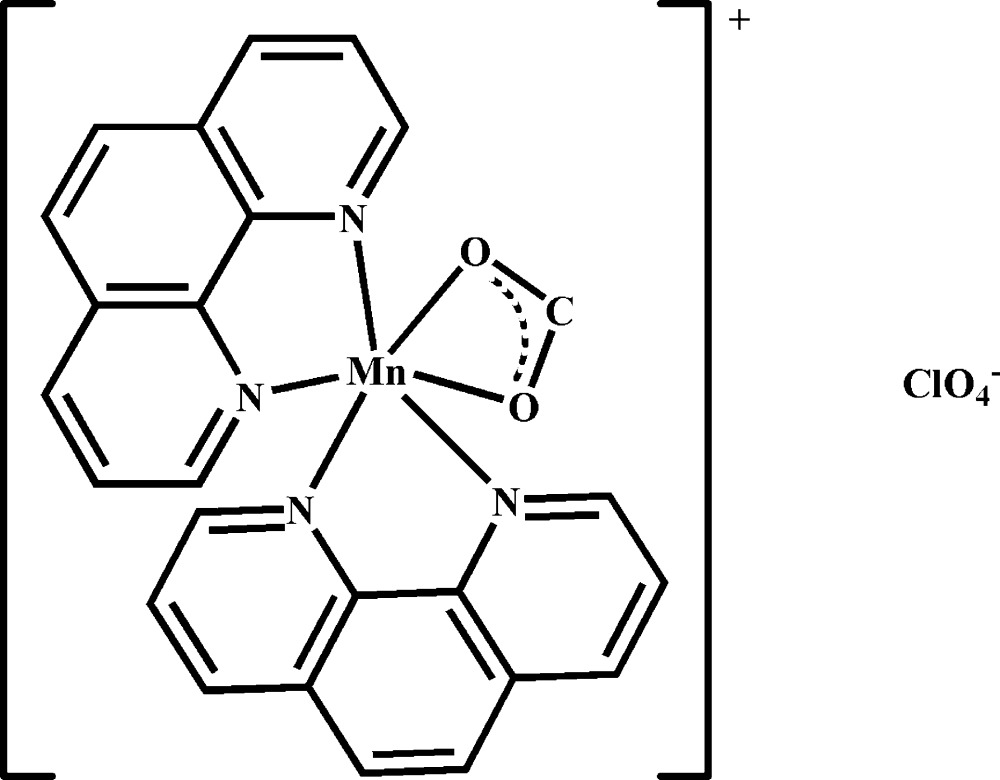


## Experimental

### 

#### Crystal data

[Mn(CHO_2_)(C_12_H_8_N_2_)_2_]ClO_4_
*M* *_r_* = 559.82
Monoclinic, 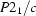

*a* = 13.0752 (10) Å
*b* = 10.9532 (9) Å
*c* = 17.4811 (14) Å
β = 111.4950 (10)°
*V* = 2329.4 (3) Å^3^
*Z* = 4
Mo *K*α radiation
μ = 0.73 mm^−1^
*T* = 293 K
0.30 × 0.25 × 0.16 mm

#### Data collection

Bruker SMART CCD diffractometer
Absorption correction: multi-scan (*SADABS*; Sheldrick, 1996 ▶) *T*_min_ = 0.803, *T*_max_ = 0.889
22324 measured reflections
5752 independent reflections
4237 reflections with *I* > 2σ(*I*)
*R*_int_ = 0.057

#### Refinement

*R*[*F* ^2^ > 2σ(*F* ^2^)] = 0.079
*wR*(*F* ^2^) = 0.176
*S* = 1.07
5752 reflections
334 parameters
2 restraints
H-atom parameters constrained
Δρ_max_ = 0.88 e Å^−3^
Δρ_min_ = −0.65 e Å^−3^

### 

Data collection: *SMART* (Bruker, 2004 ▶); cell refinement: *SAINT* (Bruker, 2004 ▶); data reduction: *SAINT*; program(s) used to solve structure: *SHELXTL* (Sheldrick, 2008 ▶); program(s) used to refine structure: *SHELXTL*; molecular graphics: *SHELXTL*; software used to prepare material for publication: *SHELXTL*.

## Supplementary Material

Crystal structure: contains datablocks I, global. DOI: 10.1107/S1600536809049277/xu2641sup1.cif

Structure factors: contains datablocks I. DOI: 10.1107/S1600536809049277/xu2641Isup2.hkl

Additional supplementary materials:  crystallographic information; 3D view; checkCIF report

## Figures and Tables

**Table 1:** Selected bond lengths (Å)

Mn1—N1	2.165 (4)
Mn1—N2	2.119 (4)
Mn1—N3	2.165 (4)
Mn1—N4	2.154 (4)
Mn1—O1	2.292 (4)
Mn1—O2	2.218 (4)

**Table 2:** Hydrogen-bond geometry (Å, °)

*D*—H⋯*A*	*D*—H	H⋯*A*	*D*⋯*A*	*D*—H⋯*A*
C2—H2⋯O1^i^	0.93	2.57	3.468 (7)	164
C5—H5⋯O4	0.93	2.43	3.355 (9)	174
C6—H6⋯O1^ii^	0.93	2.42	3.254 (7)	149
C18—H18⋯O2^iii^	0.93	2.54	3.250 (6)	134

Symmetry codes: (i) 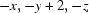; (ii) 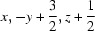; (iii) 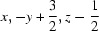.

**Fig. 1.**
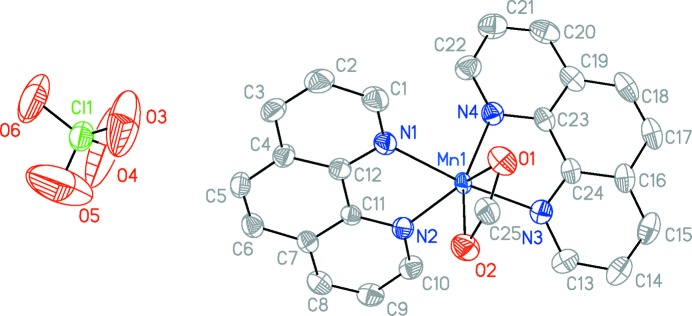
The structure of (I) with the atom-numbering scheme showing displacement ellipsoids at the 30% probability level.

**Fig. 2.**
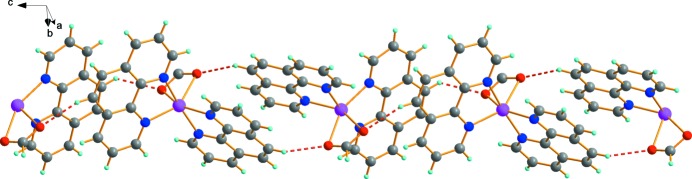
One-dimensional chain connected by C—H···O hydrogen bonds.

**Fig. 3.**
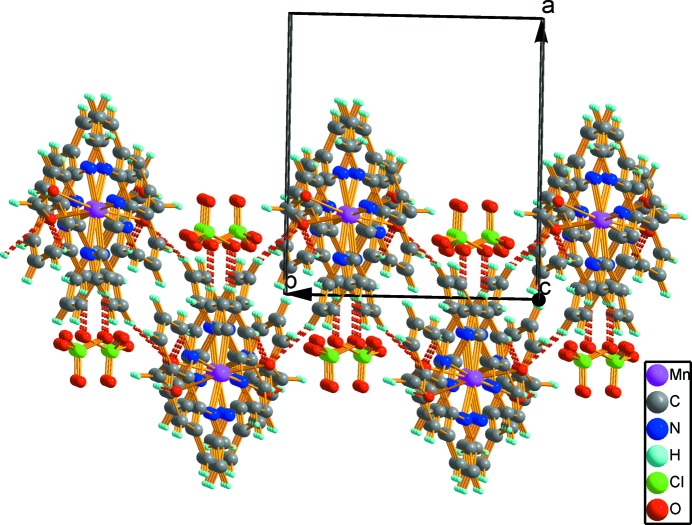
Supramolecular network formed by hydrogen-bonding and π-π stacking.

## supplementary crystallographic information

### Comment

1,10-Phenanthroline (phen), which is the parent of an important class of chelating agents, has been widely used in the construction of supramolecular architectures. Some manganese(II)-phen complexes have been synthesized and reported (Zhu *et al.*,2008; Hao *et al.*, 2008; Zhang *et al.*, 2004; Xu *et al.*, 2005). As a continuation of these studies, we herein report the crystal structure of the title complex (I).

As illustrated in Fig. 1, Mn^II^ ion is in a distorted octahedral geometry formed by two phen ligands and one HCOO^-^ anion (Table 1). The dihedral angle of two phen ligands of the complex is 57.48 (11)°. In the crystal structure the weak C—H···O hydrogen bonding links the complex into a one-dimensional chains (Fig. 2). The C2—H2···O1^i^ (symmetry code: i -x,-y + 2,-z) hydrogen bond provides additional attractive forces between adjacent chains (Table 2). Furthermore aromatic π-π stacking between N2-pyridine and C18^ii^-benzene rings [symmetry code: (ii) x, 3/2-y, 1/2+z; centroids distance = 3.656 (3) Å] helps to form the two-dimensional supramolecular motif (Fig. 3).

### Experimental

Mn(ClO_4_)_2_.6H_2_O (0.0331 g, 0.1 mmol), phen (0.0198 g, 0.1 mmol), formic acid (2 ml) and water (10 ml) were placed in a 25 ml Teflon-lined stainless steel reactor and heated at 393 K for three days, and then cooled slowly to room temperature. Single crystals were obtained from the reaction mixture.

### Refinement

All H atoms were positioned geometrically (C—H = 0.93 Å) and allowed to ride on their parent atoms, with *U*_iso_(H) = 1.2*U*_eq_(C).

### Crystal data

**Table:**

[Mn(CHO_2_)(C_12_H_8_N_2_)_2_]ClO_4_	*F*(000) = 1140
*M**_r_* = 559.82	*D*_x_ = 1.596 Mg m^−^^3^
Monoclinic, *P*2_1_/*c*	Mo *K*α radiation, λ = 0.71073 Å
Hall symbol: -P 2ybc	Cell parameters from 5752 reflections
*a* = 13.0752 (10) Å	θ = 2.2–28.3°
*b* = 10.9532 (9) Å	µ = 0.73 mm^−^^1^
*c* = 17.4811 (14) Å	*T* = 293 K
β = 111.495 (1)°	Prism, pink
*V* = 2329.4 (3) Å^3^	0.30 × 0.25 × 0.16 mm
*Z* = 4	

### Data collection

**Table:**

Bruker SMART CCD diffractometer	5752 independent reflections
Radiation source: fine-focus sealed tube	4237 reflections with *I* > 2σ(*I*)
graphite	*R*_int_ = 0.057
φ and ω scans	θ_max_ = 28.3°, θ_min_ = 2.2°
Absorption correction: multi-scan (*SADABS*; Sheldrick, 1996)	*h* = −17→12
*T*_min_ = 0.803, *T*_max_ = 0.889	*k* = −14→14
22324 measured reflections	*l* = −23→23

### Refinement

**Table:**

Refinement on *F*^2^	Primary atom site location: structure-invariant direct methods
Least-squares matrix: full	Secondary atom site location: difference Fourier map
*R*[*F*^2^ > 2σ(*F*^2^)] = 0.079	Hydrogen site location: inferred from neighbouring sites
*wR*(*F*^2^) = 0.176	H-atom parameters constrained
*S* = 1.07	*w* = 1/[σ^2^(*F*_o_^2^) + (0.0272*P*)^2^ + 7.2414*P*] where *P* = (*F*_o_^2^ + 2*F*_c_^2^)/3
5752 reflections	(Δ/σ)_max_ = 0.002
334 parameters	Δρ_max_ = 0.88 e Å^−^^3^
2 restraints	Δρ_min_ = −0.65 e Å^−^^3^

### Special details

**Table:**

Geometry. All e.s.d.'s (except the e.s.d. in the dihedral angle between two l.s. planes) are estimated using the full covariance matrix. The cell e.s.d.'s are taken into account individually in the estimation of e.s.d.'s in distances, angles and torsion angles; correlations between e.s.d.'s in cell parameters are only used when they are defined by crystal symmetry. An approximate (isotropic) treatment of cell e.s.d.'s is used for estimating e.s.d.'s involving l.s. planes.
Refinement. Refinement of *F*^2^ against ALL reflections. The weighted *R*-factor *wR* and goodness of fit *S* are based on *F*^2^, conventional *R*-factors *R* are based on *F*, with *F* set to zero for negative *F*^2^. The threshold expression of *F*^2^ > σ(*F*^2^) is used only for calculating *R*-factors(gt) *etc*. and is not relevant to the choice of reflections for refinement. *R*-factors based on *F*^2^ are statistically about twice as large as those based on *F*, and *R*- factors based on ALL data will be even larger.

### Fractional atomic coordinates and isotropic or equivalent isotropic displacement parameters (Å^2^)

**Table:**

	*x*	*y*	*z*	*U*_iso_*/*U*_eq_	
Mn1	0.28574 (5)	0.75685 (6)	0.09973 (4)	0.03794 (18)	
C12	0.1291 (4)	0.7365 (4)	0.1859 (3)	0.0485 (11)	
N3	0.4324 (3)	0.7221 (3)	0.0732 (2)	0.0513 (10)	
N4	0.2276 (3)	0.6351 (4)	−0.0041 (2)	0.0529 (10)	
C7	0.2343 (4)	0.6349 (4)	0.3166 (3)	0.0496 (11)	
C23	0.3015 (4)	0.6173 (4)	−0.0406 (3)	0.0493 (11)	
C11	0.2290 (4)	0.6786 (4)	0.2398 (3)	0.0442 (10)	
N2	0.3142 (3)	0.6696 (4)	0.2139 (2)	0.0507 (10)	
N1	0.1283 (3)	0.7782 (4)	0.1127 (2)	0.0530 (10)	
C24	0.4105 (4)	0.6658 (4)	−0.0005 (3)	0.0486 (11)	
C6	0.1396 (5)	0.6481 (5)	0.3396 (3)	0.0595 (13)	
H6	0.1420	0.6168	0.3897	0.071*	
C16	0.4886 (5)	0.6533 (5)	−0.0370 (3)	0.0582 (13)	
C10	0.4052 (4)	0.6172 (5)	0.2638 (3)	0.0610 (14)	
H10	0.4641	0.6098	0.2463	0.073*	
C8	0.3318 (5)	0.5804 (5)	0.3676 (3)	0.0632 (14)	
H8	0.3383	0.5501	0.4189	0.076*	
C4	0.0416 (4)	0.7523 (5)	0.2128 (3)	0.0540 (12)	
C3	−0.0515 (4)	0.8147 (6)	0.1588 (4)	0.0683 (15)	
H3	−0.1117	0.8282	0.1739	0.082*	
C2	−0.0533 (5)	0.8549 (6)	0.0853 (4)	0.0741 (17)	
H2	−0.1145	0.8957	0.0495	0.089*	
C5	0.0484 (5)	0.7044 (5)	0.2905 (3)	0.0617 (14)	
H5	−0.0112	0.7124	0.3071	0.074*	
C19	0.2753 (5)	0.5548 (5)	−0.1155 (3)	0.0603 (14)	
C15	0.5929 (5)	0.7011 (5)	0.0048 (4)	0.0753 (18)	
H15	0.6467	0.6964	−0.0182	0.090*	
C13	0.5328 (5)	0.7650 (5)	0.1118 (4)	0.0645 (14)	
H13	0.5487	0.8034	0.1623	0.077*	
C22	0.1285 (5)	0.5872 (5)	−0.0392 (4)	0.0679 (15)	
H22	0.0778	0.5983	−0.0139	0.082*	
C9	0.4173 (5)	0.5725 (5)	0.3409 (3)	0.0673 (15)	
H9	0.4833	0.5374	0.3742	0.081*	
C17	0.4584 (6)	0.5935 (5)	−0.1153 (4)	0.0717 (17)	
H17	0.5093	0.5873	−0.1409	0.086*	
C1	0.0378 (5)	0.8345 (6)	0.0640 (3)	0.0686 (16)	
H1	0.0353	0.8616	0.0130	0.082*	
C20	0.1692 (6)	0.5062 (5)	−0.1501 (4)	0.0750 (18)	
H20	0.1485	0.4633	−0.1994	0.090*	
C18	0.3560 (6)	0.5459 (5)	−0.1523 (3)	0.0735 (18)	
H18	0.3381	0.5067	−0.2028	0.088*	
C14	0.6158 (5)	0.7550 (6)	0.0797 (4)	0.0763 (17)	
H14	0.6860	0.7846	0.1090	0.092*	
C21	0.0962 (6)	0.5213 (6)	−0.1121 (4)	0.0781 (18)	
H21	0.0260	0.4881	−0.1344	0.094*	
Cl1	−0.21638 (13)	0.81668 (17)	0.34147 (10)	0.0761 (5)	
O2	0.3515 (3)	0.9326 (3)	0.1610 (2)	0.0695 (10)	
O1	0.2508 (3)	0.9390 (4)	0.0303 (2)	0.0709 (11)	
C25	0.3023 (5)	0.9903 (5)	0.0963 (3)	0.0607 (13)	
H25A	0.3047	1.0752	0.0977	0.073*	
O6	−0.3243 (5)	0.8154 (9)	0.3342 (5)	0.193 (4)	
O3	−0.1958 (7)	0.8782 (9)	0.2849 (6)	0.243 (6)	
O4	−0.1710 (10)	0.7089 (8)	0.3465 (11)	0.341 (10)	
O5	−0.1664 (12)	0.856 (2)	0.4119 (7)	0.436 (12)	

### Atomic displacement parameters (Å^2^)

**Table:**

	*U*^11^	*U*^22^	*U*^33^	*U*^12^	*U*^13^	*U*^23^
Mn1	0.0414 (4)	0.0420 (3)	0.0373 (3)	0.0022 (3)	0.0225 (3)	−0.0034 (3)
C12	0.048 (3)	0.048 (3)	0.049 (2)	−0.006 (2)	0.018 (2)	−0.008 (2)
N3	0.054 (3)	0.044 (2)	0.059 (2)	0.0012 (18)	0.023 (2)	−0.0032 (18)
N4	0.055 (3)	0.053 (2)	0.053 (2)	−0.003 (2)	0.022 (2)	−0.0008 (19)
C7	0.058 (3)	0.042 (2)	0.048 (2)	−0.002 (2)	0.020 (2)	−0.002 (2)
C23	0.066 (3)	0.039 (2)	0.048 (2)	0.005 (2)	0.027 (2)	0.0051 (19)
C11	0.050 (3)	0.040 (2)	0.044 (2)	−0.004 (2)	0.019 (2)	−0.0073 (18)
N2	0.046 (2)	0.052 (2)	0.055 (2)	0.0070 (19)	0.0199 (19)	−0.0019 (19)
N1	0.051 (2)	0.060 (3)	0.047 (2)	0.0073 (19)	0.0167 (19)	0.0043 (18)
C24	0.064 (3)	0.039 (2)	0.051 (3)	0.008 (2)	0.030 (2)	0.006 (2)
C6	0.070 (4)	0.061 (3)	0.053 (3)	−0.009 (3)	0.029 (3)	−0.001 (2)
C16	0.071 (4)	0.046 (3)	0.072 (3)	0.011 (3)	0.043 (3)	0.012 (2)
C10	0.051 (3)	0.059 (3)	0.072 (3)	0.012 (3)	0.022 (3)	−0.004 (3)
C8	0.075 (4)	0.055 (3)	0.054 (3)	0.007 (3)	0.017 (3)	0.005 (2)
C4	0.045 (3)	0.059 (3)	0.060 (3)	−0.003 (2)	0.021 (2)	−0.010 (2)
C3	0.045 (3)	0.084 (4)	0.074 (4)	0.003 (3)	0.019 (3)	−0.013 (3)
C2	0.051 (3)	0.092 (5)	0.068 (4)	0.022 (3)	0.010 (3)	0.001 (3)
C5	0.057 (3)	0.073 (4)	0.065 (3)	−0.009 (3)	0.034 (3)	−0.009 (3)
C19	0.086 (4)	0.046 (3)	0.049 (3)	0.010 (3)	0.024 (3)	0.001 (2)
C15	0.074 (4)	0.060 (3)	0.113 (5)	0.011 (3)	0.060 (4)	0.015 (4)
C13	0.056 (3)	0.057 (3)	0.078 (4)	−0.004 (3)	0.023 (3)	−0.007 (3)
C22	0.062 (4)	0.064 (3)	0.076 (4)	−0.010 (3)	0.023 (3)	−0.009 (3)
C9	0.061 (4)	0.066 (3)	0.063 (3)	0.019 (3)	0.009 (3)	0.009 (3)
C17	0.102 (5)	0.063 (3)	0.072 (4)	0.016 (3)	0.058 (4)	0.010 (3)
C1	0.065 (4)	0.083 (4)	0.054 (3)	0.019 (3)	0.017 (3)	0.011 (3)
C20	0.098 (5)	0.054 (3)	0.058 (3)	−0.005 (3)	0.012 (3)	−0.013 (3)
C18	0.120 (6)	0.057 (3)	0.054 (3)	0.021 (4)	0.045 (4)	0.006 (3)
C14	0.054 (3)	0.069 (4)	0.109 (5)	−0.002 (3)	0.035 (3)	0.001 (4)
C21	0.079 (5)	0.067 (4)	0.078 (4)	−0.014 (3)	0.017 (4)	−0.014 (3)
Cl1	0.0668 (10)	0.0934 (11)	0.0773 (10)	0.0128 (9)	0.0371 (8)	0.0185 (9)
O2	0.080 (3)	0.062 (2)	0.062 (2)	−0.0046 (19)	0.021 (2)	−0.0075 (17)
O1	0.088 (3)	0.066 (2)	0.058 (2)	0.009 (2)	0.026 (2)	0.0048 (17)
C25	0.067 (4)	0.062 (3)	0.056 (3)	0.005 (3)	0.026 (3)	−0.001 (3)
O6	0.095 (5)	0.290 (10)	0.239 (8)	0.044 (6)	0.111 (6)	0.058 (8)
O3	0.218 (9)	0.309 (11)	0.290 (10)	0.137 (8)	0.198 (9)	0.221 (9)
O4	0.315 (13)	0.132 (7)	0.77 (3)	0.072 (8)	0.431 (18)	0.115 (12)
O5	0.305 (17)	0.73 (4)	0.158 (9)	0.032 (19)	−0.046 (10)	−0.180 (15)

### Geometric parameters (Å, °)

**Table:**

Mn1—N1	2.165 (4)	C4—C5	1.428 (7)
Mn1—N2	2.119 (4)	C3—C2	1.349 (8)
Mn1—N3	2.165 (4)	C3—H3	0.9300
Mn1—N4	2.154 (4)	C2—C1	1.390 (8)
Mn1—O1	2.292 (4)	C2—H2	0.9300
Mn1—O2	2.218 (4)	C5—H5	0.9300
C12—N1	1.354 (6)	C19—C20	1.400 (8)
C12—C4	1.398 (6)	C19—C18	1.426 (8)
C12—C11	1.447 (6)	C15—C14	1.366 (9)
N3—C13	1.323 (7)	C15—H15	0.9300
N3—C24	1.362 (6)	C13—C14	1.397 (8)
N4—C22	1.323 (7)	C13—H13	0.9300
N4—C23	1.354 (6)	C22—C21	1.388 (8)
C7—C11	1.402 (6)	C22—H22	0.9300
C7—C8	1.394 (7)	C9—H9	0.9300
C7—C6	1.443 (7)	C17—C18	1.360 (9)
C23—C19	1.404 (6)	C17—H17	0.9300
C23—C24	1.439 (7)	C1—H1	0.9300
C11—N2	1.352 (6)	C20—C21	1.358 (9)
N2—C10	1.321 (6)	C20—H20	0.9300
N1—C1	1.329 (6)	C18—H18	0.9300
C24—C16	1.396 (7)	C14—H14	0.9300
C6—C5	1.338 (7)	C21—H21	0.9300
C6—H6	0.9300	Cl1—O5	1.240 (10)
C16—C15	1.392 (8)	Cl1—O3	1.304 (6)
C16—C17	1.436 (8)	Cl1—O4	1.310 (8)
C10—C9	1.387 (7)	Cl1—O6	1.369 (6)
C10—H10	0.9300	O2—C25	1.249 (6)
C8—C9	1.363 (8)	O1—C25	1.237 (6)
C8—H8	0.9300	C25—H25A	0.9300
C4—C3	1.414 (7)		
			
N2—Mn1—N4	113.60 (16)	C12—C4—C5	120.1 (5)
N2—Mn1—N1	78.20 (15)	C3—C4—C5	123.4 (5)
N4—Mn1—N1	95.73 (16)	C2—C3—C4	120.2 (5)
N2—Mn1—N3	105.00 (15)	C2—C3—H3	119.9
N4—Mn1—N3	77.15 (15)	C4—C3—H3	119.9
N1—Mn1—N3	172.86 (15)	C3—C2—C1	119.0 (5)
N2—Mn1—O2	91.90 (15)	C3—C2—H2	120.5
N4—Mn1—O2	154.06 (15)	C1—C2—H2	120.5
N1—Mn1—O2	94.05 (16)	C6—C5—C4	120.7 (5)
N3—Mn1—O2	92.23 (15)	C6—C5—H5	119.7
N2—Mn1—O1	145.38 (14)	C4—C5—H5	119.7
N4—Mn1—O1	98.79 (15)	C23—C19—C20	117.0 (5)
N1—Mn1—O1	87.04 (15)	C23—C19—C18	119.1 (6)
N3—Mn1—O1	93.45 (15)	C20—C19—C18	124.0 (5)
O2—Mn1—O1	57.78 (14)	C16—C15—C14	119.6 (6)
N1—C12—C4	123.5 (5)	C16—C15—H15	120.2
N1—C12—C11	117.1 (4)	C14—C15—H15	120.2
C4—C12—C11	119.3 (4)	N3—C13—C14	122.6 (6)
C13—N3—C24	118.0 (5)	N3—C13—H13	118.7
C13—N3—Mn1	128.3 (4)	C14—C13—H13	118.7
C24—N3—Mn1	112.8 (3)	N4—C22—C21	123.3 (6)
C22—N4—C23	118.1 (5)	N4—C22—H22	118.3
C22—N4—Mn1	128.3 (4)	C21—C22—H22	118.3
C23—N4—Mn1	113.2 (3)	C8—C9—C10	119.7 (5)
C11—C7—C8	118.2 (5)	C8—C9—H9	120.2
C11—C7—C6	119.1 (5)	C10—C9—H9	120.2
C8—C7—C6	122.7 (5)	C18—C17—C16	120.5 (5)
N4—C23—C19	122.5 (5)	C18—C17—H17	119.7
N4—C23—C24	117.9 (4)	C16—C17—H17	119.7
C19—C23—C24	119.6 (5)	N1—C1—C2	123.5 (5)
N2—C11—C7	122.6 (4)	N1—C1—H1	118.2
N2—C11—C12	118.1 (4)	C2—C1—H1	118.2
C7—C11—C12	119.3 (4)	C21—C20—C19	120.3 (5)
C10—N2—C11	117.6 (4)	C21—C20—H20	119.8
C10—N2—Mn1	128.5 (4)	C19—C20—H20	119.8
C11—N2—Mn1	113.7 (3)	C17—C18—C19	121.4 (5)
C1—N1—C12	117.3 (5)	C17—C18—H18	119.3
C1—N1—Mn1	129.8 (4)	C19—C18—H18	119.3
C12—N1—Mn1	112.6 (3)	C15—C14—C13	119.3 (6)
N3—C24—C16	122.8 (5)	C15—C14—H14	120.3
N3—C24—C23	117.1 (4)	C13—C14—H14	120.3
C16—C24—C23	120.1 (5)	C20—C21—C22	118.8 (6)
C5—C6—C7	121.4 (5)	C20—C21—H21	120.6
C5—C6—H6	119.3	C22—C21—H21	120.6
C7—C6—H6	119.3	O5—Cl1—O3	113.1 (12)
C15—C16—C24	117.6 (5)	O5—Cl1—O4	100.6 (11)
C15—C16—C17	123.1 (5)	O3—Cl1—O4	107.7 (6)
C24—C16—C17	119.2 (6)	O5—Cl1—O6	104.3 (8)
N2—C10—C9	123.3 (5)	O3—Cl1—O6	115.3 (5)
N2—C10—H10	118.3	O4—Cl1—O6	115.0 (6)
C9—C10—H10	118.3	C25—O2—Mn1	91.2 (3)
C9—C8—C7	118.7 (5)	C25—O1—Mn1	88.2 (3)
C9—C8—H8	120.7	O1—C25—O2	122.6 (5)
C7—C8—H8	120.7	O1—C25—H25A	118.7
C12—C4—C3	116.5 (5)	O2—C25—H25A	118.7
			
N2—Mn1—N3—C13	−68.4 (5)	Mn1—N3—C24—C23	−11.8 (5)
N4—Mn1—N3—C13	−179.7 (5)	N4—C23—C24—N3	2.5 (6)
N1—Mn1—N3—C13	175.8 (11)	C19—C23—C24—N3	−178.0 (4)
O2—Mn1—N3—C13	24.2 (5)	N4—C23—C24—C16	−177.9 (4)
O1—Mn1—N3—C13	82.1 (5)	C19—C23—C24—C16	1.6 (7)
N2—Mn1—N3—C24	123.4 (3)	C11—C7—C6—C5	−2.1 (7)
N4—Mn1—N3—C24	12.1 (3)	C8—C7—C6—C5	177.8 (5)
N1—Mn1—N3—C24	7.6 (14)	N3—C24—C16—C15	0.0 (7)
O2—Mn1—N3—C24	−144.0 (3)	C23—C24—C16—C15	−179.5 (4)
O1—Mn1—N3—C24	−86.1 (3)	N3—C24—C16—C17	−179.5 (4)
N2—Mn1—N4—C22	75.1 (5)	C23—C24—C16—C17	1.0 (7)
N1—Mn1—N4—C22	−4.5 (5)	C11—N2—C10—C9	−0.6 (8)
N3—Mn1—N4—C22	176.0 (5)	Mn1—N2—C10—C9	173.9 (4)
O2—Mn1—N4—C22	−116.2 (5)	C11—C7—C8—C9	0.2 (7)
O1—Mn1—N4—C22	−92.4 (5)	C6—C7—C8—C9	−179.7 (5)
N2—Mn1—N4—C23	−111.8 (3)	N1—C12—C4—C3	0.4 (7)
N1—Mn1—N4—C23	168.6 (3)	C11—C12—C4—C3	177.0 (4)
N3—Mn1—N4—C23	−10.8 (3)	N1—C12—C4—C5	179.3 (5)
O2—Mn1—N4—C23	57.0 (5)	C11—C12—C4—C5	−4.1 (7)
O1—Mn1—N4—C23	80.7 (3)	C12—C4—C3—C2	0.5 (8)
C22—N4—C23—C19	2.7 (7)	C5—C4—C3—C2	−178.3 (6)
Mn1—N4—C23—C19	−171.2 (4)	C4—C3—C2—C1	−0.3 (9)
C22—N4—C23—C24	−177.8 (4)	C7—C6—C5—C4	0.7 (8)
Mn1—N4—C23—C24	8.3 (5)	C12—C4—C5—C6	2.5 (8)
C8—C7—C11—N2	0.1 (7)	C3—C4—C5—C6	−178.7 (5)
C6—C7—C11—N2	−180.0 (4)	N4—C23—C19—C20	−2.6 (7)
C8—C7—C11—C12	−179.5 (4)	C24—C23—C19—C20	177.9 (5)
C6—C7—C11—C12	0.4 (7)	N4—C23—C19—C18	176.5 (4)
N1—C12—C11—N2	−0.2 (6)	C24—C23—C19—C18	−3.0 (7)
C4—C12—C11—N2	−177.0 (4)	C24—C16—C15—C14	1.6 (8)
N1—C12—C11—C7	179.4 (4)	C17—C16—C15—C14	−178.9 (5)
C4—C12—C11—C7	2.7 (7)	C24—N3—C13—C14	0.2 (8)
C7—C11—N2—C10	0.1 (7)	Mn1—N3—C13—C14	−167.4 (4)
C12—C11—N2—C10	179.7 (4)	C23—N4—C22—C21	−0.9 (8)
C7—C11—N2—Mn1	−175.2 (3)	Mn1—N4—C22—C21	172.0 (4)
C12—C11—N2—Mn1	4.4 (5)	C7—C8—C9—C10	−0.7 (8)
N4—Mn1—N2—C10	89.4 (5)	N2—C10—C9—C8	0.9 (9)
N1—Mn1—N2—C10	−179.5 (5)	C15—C16—C17—C18	178.4 (5)
N3—Mn1—N2—C10	7.1 (5)	C24—C16—C17—C18	−2.1 (8)
O2—Mn1—N2—C10	−85.8 (4)	C12—N1—C1—C2	1.7 (9)
O1—Mn1—N2—C10	−112.8 (5)	Mn1—N1—C1—C2	−171.3 (5)
N4—Mn1—N2—C11	−96.0 (3)	C3—C2—C1—N1	−0.9 (10)
N1—Mn1—N2—C11	−4.8 (3)	C23—C19—C20—C21	0.7 (8)
N3—Mn1—N2—C11	−178.3 (3)	C18—C19—C20—C21	−178.3 (6)
O2—Mn1—N2—C11	88.9 (3)	C16—C17—C18—C19	0.7 (9)
O1—Mn1—N2—C11	61.9 (4)	C23—C19—C18—C17	1.9 (8)
C4—C12—N1—C1	−1.5 (7)	C20—C19—C18—C17	−179.0 (6)
C11—C12—N1—C1	−178.2 (5)	C16—C15—C14—C13	−2.2 (9)
C4—C12—N1—Mn1	172.7 (4)	N3—C13—C14—C15	1.4 (9)
C11—C12—N1—Mn1	−4.0 (5)	C19—C20—C21—C22	0.9 (9)
N2—Mn1—N1—C1	178.0 (5)	N4—C22—C21—C20	−0.9 (10)
N4—Mn1—N1—C1	−69.1 (5)	N2—Mn1—O2—C25	−159.4 (3)
N3—Mn1—N1—C1	−64.7 (14)	N4—Mn1—O2—C25	30.9 (5)
O2—Mn1—N1—C1	86.9 (5)	N1—Mn1—O2—C25	−81.1 (3)
O1—Mn1—N1—C1	29.5 (5)	N3—Mn1—O2—C25	95.5 (3)
N2—Mn1—N1—C12	4.7 (3)	O1—Mn1—O2—C25	2.8 (3)
N4—Mn1—N1—C12	117.7 (3)	N2—Mn1—O1—C25	29.6 (5)
N3—Mn1—N1—C12	122.1 (12)	N4—Mn1—O1—C25	−170.8 (3)
O2—Mn1—N1—C12	−86.4 (3)	N1—Mn1—O1—C25	93.8 (3)
O1—Mn1—N1—C12	−143.8 (3)	N3—Mn1—O1—C25	−93.3 (3)
C13—N3—C24—C16	−0.9 (7)	O2—Mn1—O1—C25	−2.9 (3)
Mn1—N3—C24—C16	168.6 (4)	Mn1—O1—C25—O2	5.1 (6)
C13—N3—C24—C23	178.6 (4)	Mn1—O2—C25—O1	−5.3 (6)

### Hydrogen-bond geometry (Å, °)

**Table:**

*D*—H···*A*	*D*—H	H···*A*	*D*···*A*	*D*—H···*A*
C2—H2···O1^i^	0.93	2.57	3.468 (7)	164
C5—H5···O4	0.93	2.43	3.355 (9)	174
C6—H6···O1^ii^	0.93	2.42	3.254 (7)	149
C18—H18···O2^iii^	0.93	2.54	3.250 (6)	134

Symmetry codes: (i) −*x*, −*y*+2, −*z*; (ii) *x*, −*y*+3/2, *z*+1/2; (iii) *x*, −*y*+3/2, *z*−1/2.
